# Injury mortality in rural South Africa 2000 – 2007: rates and associated factors*

**DOI:** 10.1111/j.1365-3156.2011.02730.x

**Published:** 2011-04

**Authors:** Anupam Garrib, Abraham J Herbst, Victoria Hosegood, Marie-Louise Newell

**Affiliations:** 1 Africa Centre for Health and Population Studies, University of KwaZulu-NatalSomkhele, South Africa; 2 Faculty of Social and Human Sciences, University of SouthamptonUK; 3 MRC Centre of Epidemiology for Child Health, University College LondonUK

**Keywords:** mortality, wounds and Injuries, rural population, South Africa, epidemiology, road traffic injuries

## Abstract

**Objective:**

To estimate injury mortality rates in a rural population in KwaZulu-Natal, South Africa and to identify socio-demographic risk factors associated with adult injury-related deaths.

**Methods:**

The study used population-based mortality data collected by a demographic surveillance system on all resident and non-resident members of 11 000 households. Deaths and person-years of observation (pyo) were aggregated for individuals between 01 January 2000 and 31 December 2007. Cause of death was determined by verbal autopsy, coded using ICD-10 and further categorised using global burden of disease categories. Socio-demographic risk factors associated with injuries were examined using regression analyses.

**Results:**

We analysed data on 133 483 individuals with 717 584.6 person-years of observation (pyo) and 11 467 deaths. Of deaths, 8.9% were because of injury-related causes; 11% occurred in children <15 years old. Homicide, road traffic injuries and suicide were the major causes. The estimated crude injury mortality rate was 142.4 (134.0, 151.4)/100 000 pyo; 116.9 (108.1, 126.5)/100 000 pyo among residents and 216.8 (196.5, 239.2)/100 000 pyo among non-residents. In multivariable analyses, the differences between residents and non-residents remained but were no longer significant for women. In men and women, full-time employment was significantly associated with lower mortality [adjusted rate ratios 0.6 (0.4, 0.9); 0.4 (0.2, 0.9)]; in men, higher asset ownership was independently associated with increased mortality [adjusted rate ratio 1.5 (1.1, 1.9)].

**Conclusions:**

Reducing the high levels of injury-related mortality in South Africa requires intersectoral primary prevention efforts that redress the root causes of violent and accidental deaths: social inequality, poverty and alcohol abuse.

## Introduction

Injuries are a major cause of morbidity and mortality in low- and middle-income countries (LMIC) (Peden *et al.* 2002). South Africa has extremely high injury mortality rates, with homicide rates six times higher than, and road traffic injury (RTI) mortality rates nearly double, global injury mortality rates (Norman *et al.* 2007a; Seedat *et al.* 2009). Although the proportion of deaths because of injuries has fallen, from 17% of all deaths in 1997 to 8.7% in 2006, this decline is partly due to improvements in reporting of deaths overall and HIV-related mortality increases (Statistics South Africa 2006). Injury mortality is concentrated among young adults; almost 50% of global injury mortality occurs in the 15- to 44-year age group (Peden *et al.* 2002; Herbst *et al.* 2009). In South Africa, young men bear a disproportionate share of the injury burden (Matzopoulos *et al.* 2008a).

Across Africa, information on injuries is mostly derived from small surveys and epidemiological studies, with few data available on injuries in rural areas. Within the category of injury deaths, causes vary across the region, but major causes are homicide, RTIs, war-related injuries, suicide, drowning, falls and burns (Bowman *et al.* 2006). Estimating the burden of injury mortality in South Africa is also limited by available data (Norman *et al.* 2007a,b;): vital registration data remain incomplete, and cause of death is poorly defined; the findings of a national injury surveillance study (NIMSS) were biased towards urban areas (Norman *et al.* 2007b). In this study, we describe injury-related mortality in a demographic surveillance system in rural KwaZulu-Natal. Demographic and socio-economic data were used to examine factors associated with injury mortality risk.

## Methods

The study was conducted at the Africa Centre's ongoing demographic surveillance site in northern KwaZulu-Natal, South Africa (Tanser *et al.* 2008).The area is typical of many South African rural areas in that while predominantly rural, it contains an urban township and an informal peri-urban settlement.

Routine surveillance visits to each household were made three times a year from 2000 to 2003 and twice a year thereafter. Key household informants provide up-to-date demographic and health information about all resident and non-resident household members, including mortality, fertility, and migration. Socio-economic data such as information about education, employment, and household wealth were obtained once a year. Non-residents are household members whose primary residences are elsewhere, for example labour migrants who stay closer to their workplace but return for visits (Muhwava *et al.* 2007).

To classify the cause of death, a nurse re-visits the household for a verbal autopsy interview with one or more informants involved in caring for the deceased or able to provide information about the circumstances of death. The verbal autopsy interview occurs on average 9 months after a death is reported at routine surveillance visits. Informants are asked to narrate the course of the illness or events leading to death. A structured interview is then administered (INDEPTH Network 2003). The injury section of the questionnaire contains detailed questions on geographical location, mechanism and intent of the injury. Information from available health records is recorded.

Interviews were conducted in the local language and transcribed by the interviewer, after verbal consent. Ethical approval for the Africa Centre Demographic Surveillance was provided by the University of KwaZulu-Natal Bio-Medical Research Ethics Committee. Verbal autopsy questionnaires were independently analysed by two physicians to attribute underlying and if possible, contributory, causes of death. A third physician coded the cause of death using ICD-10. Where there was no agreement on the cause of death, the third doctor arranged a consensus meeting; if no agreement was reached, the cause was assigned as ‘undetermined’, usually when there was inadequate information, no informant could be found, or consent for the interview was refused. ICD-10 codes were further classified into global burden of disease categories (Mathers *et al.* 2008).

In the analyses presented here, deaths and person-years of observation were aggregated per year for the period 1 January 2000 to 31 December 2007 for all individuals in the study population. Individuals contributed to the person-years denominator from 1 January 2000, or from any later date of birth or in-migration, and ceased to contribute to the denominator at death, termination of household membership, household out-migration or the last surveillance visit in which household membership was confirmed. Mortality rates by sex, year and age group were calculated for all injury deaths and for the three most commonly attributed causes of injury mortality.

Multivariable Poisson regression was used to identify factors associated with survival in adults 15 years and older only. Risk factors were modelled separately for men and women given anticipated differences in risk of death by gender (Krug *et al.* 2002; Seedat *et al.* 2009). Factors associated with injury mortality in children were not explored further because limited data about parental and caregiver characteristics were available (Howe *et al.* 2006). Factors considered were age, education, employment, household socio-economic status, migration and area of residence.

Education was classified based on the highest level of education attained, or current level if the individual was still in education. Migration status was classified into those who had always lived within the surveillance area, those who had always lived outside of the area and those who had migrated either in or out. A household asset index was created to indicate socio-economic status using an asset summation method (Case *et al.* 2005). Area of residence was classified into rural, peri-urban and urban based on settlement density; non-residents were categorised as non-resident. Demographic and socio-economic data collected closest prior to death were used in this analysis. Models were compared using the likelihood ratio test. All analyses were performed using STATA v 11 (STATA Co, College station, TX, USA).

## Results

A total of 11 467 deaths were recorded for 717 584.6 person-years of observation (pyo) in 133 483 people giving an overall mortality rate of 1598.0 deaths per 100 000 person-years. Of these deaths, 1022 (8.9%) were attributed to injury-related causes, with an injury mortality rate of 142.4 per 100 000 pyo. Most injury deaths occurred in men (*N* = 787/1022, 77%). Injury mortality was highest in young adults: 31% (316/1022) of all injury deaths occurred in the 20- to 29-year age group (Table 1) and 11% (117/1022) in children younger than 15 years.

**Table 1:** All deaths and injury deaths by age group and sex, Africa Centre 2000–2007

	All deaths (2000–2007)		Injury deaths (2000–2007)	
		
	Male	Female	Male	Female
				
Age group (years)	*N*	*N*	*N* (Proportion of all male deaths in age group, %)	*N* (Proportion of all female deaths in age group, %)
0–4	764	698	23 (3.0)	24 (3.4)
5–9	92	96	20 (21.7)	16 (16.7)
10–14	55	58	16 (29.1)	16 (27.6)
15–19	107	127	55 (51.4)	18 (14.1)
20–29	832	1151	267 (32.1)	49 (4.3)
30–39	1362	1249	194 (14.2)	20 (1.6)
40–49	989	705	100 (10.1)	27 (3.8)
50–59	588	404	50 (8.5)	18 (4.5)
60–69	467	429	34 (7.3)	22 (5.1)
70–79	334	431	23 (6.9)	18 (4.2)
80+	199	329	5 (2.5)	7 (2.1)
Total	5790	5677	787 (13.6)	235 (4.1)

### Causes of death

**Figure 1:**
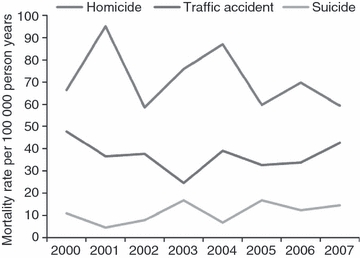
Mortality rates per 100 000 person-years for all homicide, traffic accident and suicide deaths by year.

Homicide is the single most common cause of injury death in both men and women; it comprises 50% (513/1022) of all injury mortality. Annual homicide rates ranged between a high of 95.2 (77.0, 117.5) deaths per 100 000 person-years in 2001 and a low of 58.6 (44.7, 76.7) deaths per 100 000 person-years in 2002 (Figure 1). For both sexes combined, 63% (325/513) of homicides were because of gunshot-related trauma and 23% (118/513) to stabbings (Table 2). There were nine homicide deaths in children under 15 years of age. Mortality rates for homicide deaths were higher in men in all age groups and peaked in the 30- to 39-year age group at 289.5 (241.0, 347.8) deaths per 100 000 person-years. In women, the homicide mortality rates peaked in the 70- to 79-year age group at 96.8 (50.4, 186.1) deaths per 100 000 person-years.

**Table 2:** Mortality rates for all injury deaths per 100 000 person years by sex and attributed cause, Africa Centre 2000–2007

	Male			Female			Total		
			
Cause of death	*N*	%	Mortality rate	*N*	%	Mortality rate	*N*	%	Mortality rate
Homicide, of which:	427	54.3	125.4 (114.1, 137.9)	86	36.6	22.8 (18.5, 28.2)	513	50.2	71.5 (65.6, 78.0)
Homicide by gunshot*	274	34.8	80.5 (71.5, 91.0)	51	21.7	13.5 (10.3, 17.8)	325	31.8	45.3 (40.6, 50.5)
Homicide by stabbing*	101	12.8	29.7 (24.4, 36.1)	17	7.2	4.5 (2.8, 7.3)	118	11.5	16.4 (13.7, 19.7)
Traffic accident, of which:	194	24.7	57.0 (49.5, 65.6)	70	29.8	18.6 (14.7, 23.5)	264	25.8	36.8 (32.6, 41.5)
Traffic accident - vehicle occupants	123	15.6	36.1 (30.3, 43.1)	42	17.9	11.1 (8.2, 15.1)	165	16.1	23.0 (19.7, 26.8)
Traffic accident - pedestrians	71	9.0	20.9 (16.5, 26.3)	28	11.9	7.4 (5.1, 10.8)	99	9.7	13.8 (11.3, 16.8)
Suicide, of which:	68	8.6	20.0 (15.7, 25.3)	13	5.5	3.4 (2.0, 5.9)	81	7.9	11.3 (9.1, 14.0)
Suicide by hanging*	47	6.0	13.8 (10.4, 18.4)	8	3.4	2.1 (1.1, 4.2)	55	5.4	7.7 (5.9, 10.0)
Other accidental death	35	4.4	10.3 (7.4, 14.3)	23	9.8	6.1 (4.1, 9.2)	58	5.7	8.1 (6.2, 10.5)
Fire	16	2.0	4.7 (2.9, 7.7)	24	10.2	6.4 (4.3, 9.5)	40	3.9	5.6 (4.1, 7.6)
Drowning	21	2.7	6.2 (4.0, 9.5)	13	5.5	3.4 (2.0, 5.9)	34	3.3	4.7 (3.4, 6.6)
Poisoning	22	2.8	6.5 (4.3, 9.8)	5	2.1	1.3 (0.6, 3.2)	27	2.6	3.8 (2.6, 5.5)
Undetermined intent	5	0.6	0.7 (0.3, 1.7)	4	1.7	1.2 (0.4, 3.1)	1	0.1	0.3 (0.04, 1.88)
All deaths	787	100	231.1 (215.5, 247.9)	235	100	62.3 (54.8, 70.8)	1022	100	142.4 (134.0, 151.4)

* Only the major causes of homicide and suicide are presented in this table.

Road traffic accidents accounted for 26% (264/1022) of all injury deaths of adults with 38% (99/264) of these deaths occurring in pedestrians. In children, RTIs were the single most common cause of injury-related mortality (38%; 45/117), the majority pedestrian deaths. The 0- to 9-year age group was the only one in which RTI deaths were more frequent than homicide. Mortality rates because of RTIs were again significantly higher in men than women in all age groups, for both pedestrians and vehicle occupant mortality. RTI mortality rates fell from 47.6 (35.3, 64.1) deaths per 100 000 person-years in 2000 to 24.6 (16.2, 37.3) in 2003, then rising again to 42.6 (31.0, 58.5) in 2007 (Figure 1).

Suicide was the third most common cause of injury death; 84% (68/81) of suicide deaths were in men. All suicides in women occurred between the ages of 10 to 40 years, and 54% (37/68) of those in men occurred in the 20–29 age group, with a mortality rate of 54.3 (39.3, 74.9) [females 5.4 (2.0, 14.4)] deaths per 100 000 person-years. Hanging (55/81, 68%) and gunshot (14/81, 17%) were the most common methods (Table 2).

### Other causes of death

Accidental drowning accounted for 3.3% (*N* = 34) of injury-related deaths, with 65% (22/34) occurring in children aged under 15 years. Drowning was the second commonest cause of injury death in children after RTIs (18.8%; 22/117). There were 34 injury-related deaths reported to have occurred at the workplace, but the attributed cause of death was homicide in 23 and RTI in 5 of these cases. Sexual violence was attributed as cause in one death. There were five deaths where it could not be determined whether the death was accidental or not. Accidental fire was the only cause of death which killed more women than men.

### Factors associated with injury death in adults

Tables 3 and 4 present mortality rates and rate ratio statistics for men and women by age, education, employment, migration, area of residence and household assets from multivariable regression analyses. In women, with increasing age, risk falls to its lowest level relative to baseline in the 30- to 39-year age group before rising again. In men, the pattern was less clear, although the association with age was statistically significant. The elderly were at the highest risk of injury death after controlling for other factors. In both men and women, no education was associated with lower risk of injury mortality. Full-time employment was associated with significantly lower injury mortality risk in men and approached statistical significance in females. Both male and female non-residents were at considerably lower risk of injury death, although not significantly so in the case of women (likely due to lack of statistical power with only 55 non-resident females but 323 non-resident males). Female residents of peri-urban areas had nearly half the risk of dying because of injury as female residents of rural areas. Men resident in urban areas were at 60% risk of injury mortality compared to men resident in rural areas. Higher levels of asset possession were also associated with an increased risk of injury mortality although only significantly so in men.

**Table 3:** Factors associated with mortality from injury among women ≥15 years

	Univariate analysis				Multivariable analysis		
		
		Mortality rate (95% CI)	Rate ratio (95% CI)	*P*-value	Rate ratio (95% CI)	*P*-value	LR test (*P*-value)
Age group							
15–19 year	18	38.3 (24.2, 60.9)	1		1		0.01
20–29 year	49	66.3 (50.1, 87.7)	1.6 (1.0, 2.8)	0.07	1.9 (1.0, 3.7)	0.07	
30–39 year	20	43.3 (28.0, 67.2)	1.1 (0.6, 2.1)	0.78	1.3 (0.6, 2.9)	0.52	
40–49 year	27	85.8 (58.9, 125.2)	2.3 (1.3, 4.2)	0.005	3.0 (1.4, 6.9)	0.01	
50–59 year	18	99.8 (62.9, 158.5)	2.7 (1.4, 5.1)	0.003	2.8 (1.0, 7.8)	0.05	
60–69 year	22	160.5 (105.7, 243.8)	4.4 (2.4, 8.2)	<0.0001	2.9 (1.0, 8.6)	0.06	
70–79 year	18	193.7 (122.0, 307.5)	5.4 (2.8, 10.4)	<0.0001	7.3 (2.8, 19.3)	<0.001	
80+ year	7	214.1 (102.1, 449.1)	6.3 (2.6, 15.1)	<0.0001	4.5 (0.9, 21.7)	0.06	
Education							
No education	11	33.8 (18.8, 61.1)	0.76 (0.4, 1.4)	0.40	0.4 (0.2, 1.0)	0.04	0.07
Primary school	30	73.3 (51.2, 104.8)	1.5 (0.9, 2.3)	0.09	0.9 (0.5, 1.6)	0.74	
Secondary school	58	47.0 (36.3, 60.8)	1		1		
Post-school education	4	41.0 (15.4, 109.3)	0.9 (0.3, 2.4)	0.80	1.1 (0.4, 3.1)	0.89	
Employed							
Unemployed	47	294.9 (221.6, 392.5)	1		1		0.10
Part-time employment	73	213.8 (169.9, 268.9)	0.7 (0.5, 1.1)	0.12	0.7 (0.4, 1.1)	0.08	
Full-time employment	9	126.6 (65.9, 243.3)	0.4 (0.2, 0.9)	0.02	0.4 (0.2, 1.0)	0.05	
Migration							
Always resident	108	87.6 (72.6, 105.8)	1		1		0.07
Migrated	30	32.4 (22.6, 46.3)	0.4 (0.3, 0.6)	<0.0001	0.7 (0.3, 1.3)	0.25	
Always non-resident	41	152.9 (112.5, 207.6)	1.6 (1.1, 2.3)	0.01	1.4 (0.5, 3.8)	0.52	
Asset index							
<12	59	114.0 (88.3, 147.1)	1		1		0.6
≥12–<16	41	67.7 (49.8, 91.9)	0.7 (0.5, 1.0)	0.05	1.5 (0.8, 2.7)	0.22	
≥16–<20	44	68.5 (50.9, 92.0)	0.7 (0.5, 1.0)	0.05	1.4 (0.8, 2.6)	0.26	
>20	35	53.6 (38.5, 74.6)	0.5 (0.3, 0.8)	0.003	1.4 (0.8, 2.6)	0.27	
Area of residence							
Rural	71	71.2 (56.5, 89.9)	1		1		<0.001
Peri-urban	40	84.2 (61.8, 114.8)	1.1 (0.8, 1.6)	0.61	0.5(0.3, 0.8)	0.01	
Urban	4	32.1 (12.0, 85.5)	0.4 (0.1, 1.0)	0.06	–*	–	
Non-resident	55	77.3 (59.4, 100.7)	1 (0.7, 1.4)	0.99	0.6(0.2, 1.4)	0.22	

* Number of cases (4) too low.

**Table 4:** Factors associated with mortality from injury among men ≥15 years

	Univariate analysis				Multivariate analysis		
		
	*N*	Mortality rate (95% CI)	Rate ratio (95% CI)	*P*-value	Rate ratio (95% CI)	*P*-value	LR test (*P*-value)
Age group							
15–19 year	55	120.4 (92.4, 156.8)	1		1		<0.0001
20–29 year	267	391.8 (347.5, 441.7)	3.1 (2.3, 4.1)	<0.0001	5.2 (3.6, 7.6)	<0.0001	
30–39 year	194	492.8 (428.1, 567.3)	3.8 (2.8, 5.1)	<0.0001	5.0 (3.4, 7.3)	<0.0001	
40–49 year	100	393.4 (323.3, 478.5)	3.2 (2.3, 4.4)	<0.0001	5.0 (3.3, 7.6)	<0.0001	
50–59 year	50	353.6 (268.0, 466.5)	2.8 (1.9, 4.1)	<0.0001	2.6 (1.4, 4.9)	0.002	
60–69 year	34	434.8 (310.7, 608.5)	3.5 (2.3, 5.3)	<0.0001	3.3 (1.7, 6.6)	<0.0001	
70–79 year	23	562.6 (373.8, 846.6)	4.6 (2.8, 7.4)	<0.0001	5.0 (2.4, 10.4)	<0.0001	
80+ year	5	364.3 (151.6, 875.3)	2.9 (1.1, 7.2)	0.02	6.3 (2.2, 18.3)	0.001	
Education							
No education	29	160.4 (111.5, 230.9)	0.7 (0.4, 1.0)	0.03	0.5 (0.3, 0.8)	0.005	0.009
Primary school	136	395.5 (334.3, 467.9)	1.4 (1.2, 1.8)	0.001	1.1 (0.8, 1.3)	0.64	
Secondary school	282	247.3 (220.1, 277.9)	1		1		
Tertiary education	16	244.3 (149.7, 398.7)	1.0 (0.6, 1.6)	0.97	0.8 (0.4, 1.3)	0.34	
Employment							
Unemployed	117	1164.9 (971.9, 1396.4)	1		1		<0.0001
Part-time employment	352	1162.3 (1047.0, 1290.3)	1.1 (0.9, 1.3)	0.50	1.3 (1.0, 1.7)	0.02	
Full-time employment	67	703.8 (553.9, 894.2)	0.6 (0.5, 0.9)	0.004	0.6 (0.4, 0.9)	0.004	
Migration							
Always resident	316	419.1 (375.4, 468.0)	1		1		0.16
Migrated	192	215.6 (187.1, 248.3)	0.6 (0.5, 0.7)	<0.0001	1.0 (0.8, 1.3)	0.82	
Always non-resident	220	529.3 (463.8, 604.0)	1.3 (1.1, 1.5)	0.003	1.3 (0.9, 1.9)	0.22	
Asset index							
<12	190	421.3 (365.5, 485.7)	1		1		0.02
≥12–<16	190	363.6 (315.4, 419.2)	0.99 (0.81, 1.21)	0.93	1.5 (1.1, 1.9)	0.007	
≥16–<20	172	323.3 (278.4, 375.4)	0.88 (0.72, 1.08)	0.22	1.4 (1.0, 1.8)	0.03	
>20	175	319.0 (275.1, 370.0)	0.89 (0.72, 1.09)	0.25	1.5 (1.1, 1.9)	0.01	
Area of residence							
Rural	202	325.2 (283.3, 373.3)	1		1		0.002
Peri-urban	168	492.0 (423.0, 572.3)	1.4 (1.2, 1.8)	0.001	1.1 (0.8, 1.4)	0.69	
Urban	20	252.9 (163.2, 392.0)	0.7 (0.4, 1.0)	0.08	0.4 (0.2, 0.8)	0.008	
Non-resident	323	347.5 (311.6, 387.6)	1.1 (0.9, 1.3)	0.44	0.7 (0.5, 1.0)	0.04	

## Discussion

We present population-based data on rates and causes of injury-related deaths from a predominantly rural area in South Africa. Although verbal autopsies have limited sensitivity and specificity for some causes of death (Soleman *et al.* 2006), injury-related deaths have a defined sequence of events that is less likely to be misclassified, and verbal autopsy data provide an accurate indication of cause specific injury mortality.

Nearly 1 in 10 of all deaths were caused by injury, with an injury mortality rate of 142.4 (134.0, 151.4) per 100 000 pyo, almost twice the 2000 global estimate of 83.7 deaths per 100 000 population (Peden *et al.* 2002). Although there is a lack of available data, it is often assumed that injury mortality rates are lower in rural than urban areas. Our estimated injury mortality rate was marginally higher than the 2007 NIMSS estimate of 134.8 per 100 000 population for Durban, the closest major city (MRC/UNISA Crime Violence and Injury Lead Programme 2008). Detailed rural statistics for KwaZulu-Natal are not available, but 2009 NIMSS estimates of 147.9 per 100 000 population from the predominantly rural Mpumulanga province are comparable to what we found here (MRC-UNISA Safety & Peace Promotion Research Unit 2010).

Mortality because of homicide in this population in rural KwaZulu-Natal was nine times higher than the global homicide mortality estimate in 2000 (Krug *et al.* 2002). The fivefold homicide rate difference between the sexes is higher than the threefold difference reported globally. The most common method of homicide was the use of firearms, reflecting the widespread availability of guns in South African society. South Africa's recent political history and marked social and economic inequalities are contributing factors to the high rates of interpersonal violence in the country (Norman *et al.* 2007a; Seedat *et al.* 2009). Particular to this area is a history of violent conflicts between different factions in the community which resulted in the deaths of several men reported in this study. In rural Mpumulanga, the two major causes of injury deaths were reversed, with road traffic accidents accounting for 45.3% of injury mortality, and homicide accounting for 22.5% (MRC-Unisa Safety & Peace Promotion Research Unit 2010).

Young men are at highest risk of injury-related mortality and constitute the majority of perpetrators as well as victims of violent incidents (Matzopoulos *et al.* 2008b; Seedat *et al.* 2009). Several other factors are associated with risk of injury mortality: poverty, lack of education, unemployment, alcohol and substance abuse, interpersonal conflict around money, intimacy and power (Norman *et al.* 2007a; Seedat *et al.* 2009). We found a sex difference in the association of education and employment factors to injury mortality risk. The pattern of injuries in this population was similar to the national data presented by Seedat *et al.* (2009) showing that male youth unemployment consistently correlated with homicide and assault (Seedat *et al.* 2009). Further, men and women who were non-resident in the rural surveillance area were at considerably lower risk of injury death univariably, although no longer significantly so in adjusted analyses in the case of women. Among women and men resident in the area, those living in peri-urban areas were at significantly lower risk of injury death than those living in more rural areas.

Our findings give further impetus to calls for intervention strategies addressing violent behaviour in young men to be accompanied by strategies to address employment and education opportunities. Furthermore, effective interventions are needed to promote responsible alcohol use and minimise access to firearms, both of which contribute significantly to the high rate of fatal and non-fatal injuries in South Africa (MRC/UNISA Crime Violence and Injury Lead Programme 2008, Seedat *et al.* 2009). Homicide and RTIs remain the predominant causes of injury deaths in older adults.

Traffic accident-related mortality was three times higher than the global rate of 13 deaths per 100 000 persons (Peden *et al.* 2004). RTIs are the leading cause of injury-related mortality in both developed and developing countries with pedestrians and young children bearing a disproportionate share of the burden (Hobday & Knight 2010a). The rise in RTI mortality in developing countries is a result of economic growth and growing numbers of motor vehicles. In contrast, RTI mortality has been declining in developed countries over the last 40 years after the introduction of legislation and safety measures and the development of public transport systems (Ameratunga *et al.* 2006; Garg & Hyder 2006).

To reduce the high levels of RTI mortality, current road safety policy in South Africa focuses on the use of seatbelts, child restraints and helmets, and combating aggressive driving and driving under the influence of alcohol. But these interventions, which are aimed at road user behaviour, have been poorly enforced (Norman *et al.* 2007a; Matzopoulos *et al.* 2008c; Seedat *et al.* 2009) and without adequate enforcement will have limited impact (O'Neill & Mohan 2002). A combination of measures is needed that addresses road user behaviour and improves both roads and vehicle design to better protect passengers and pedestrians (Peden *et al.* 2008). Children are at particular risk of RTI death as they are unable to make safe decisions and appropriately judge risk on the road (Peden *et al.* 2008). In the study area, structural and environmental interventions that separate pedestrians and vehicles, reduce traffic speeds and create safe road crossings are needed, particularly around schools, playgrounds and commercial areas (Matzopoulos *et al.* 2008c; Hobday & Knight 2010a,b;). A more detailed examination of the circumstances of RTI deaths in the area could also provide more concrete information on underlying causes and contributory factors and inform interventions.

In a population already experiencing high levels of AIDS mortality, the burden of child and adult injuries has potentially severe social and economic consequences for households (Hosegood *et al.* 2004a). Households experiencing a violent or accidental adult death are at more than twice the risk of dissolving as households experiencing a death from any other cause, reflecting the social consequences of injury mortality (Hosegood *et al.* 2004b). HIV-related mortality accounted for 71.5% of deaths in the 25- to 49-year age group, with declines after the HIV treatment roll-out (Herbst *et al.* 2009). As the HIV treatment programme continues to expand, injuries are likely to become a more prominent contributor to the mortality burden in the young adult population.

Primary prevention of the injury burden involves addressing the social inequality, unemployment and poverty root factors (Butchart & Engstrom 2002; Norman *et al.* 2007a; Matzopoulos *et al.* 2008a; Seedat *et al.* 2009). This will require economic development and long-term social change that can only follow concerted action from government and civil society. An evidence-based approach to injury control is crucial, and its implementation needs recognition of the public health challenge presented by injuries, appropriate resource allocation and adequate monitoring of the impact of interventions (Mock 2001; Seedat *et al.* 2009). This study contributes population-based longitudinal data to improve our knowledge of the injury health burden in South Africa.
